# Bacteriophage endolysins as a potential weapon to combat *Clostridioides difficile* infection

**DOI:** 10.1080/19490976.2020.1813533

**Published:** 2020-09-28

**Authors:** Shakhinur Islam Mondal, Lorraine A. Draper, R Paul Ross, Colin Hill

**Affiliations:** a APC Microbiome Ireland, University College Cork, Cork, Ireland; b Genetic Engineering and Biotechnology Department, Shahjalal University of Science and Technology, Sylhet, Bangladesh; c School of Microbiology, University College Cork, Cork, Ireland; d Teagasc Food Research Centre, Moorepark, Cork, Ireland

**Keywords:** *Clostridioides difficile*, endolysin; bacteriophage, antimicrobials, *clostridioides difficile* infections, antibiotics, novel therapy

## Abstract

*Clostridioides difficile* is the leading cause of health-care-associated infection throughout the developed world and contributes significantly to patient morbidity and mortality. Typically, antibiotics are used for the primary treatment of *C. difficile* infections (CDIs), but they are not universally effective for all ribotypes and can result in antibiotic resistance and recurrent infection, while also disrupting the microbiota. Novel targeted therapeutics are urgently needed to combat CDI. Bacteriophage-derived endolysins are required to disrupt the bacterial cell wall of their target bacteria and are possible alternatives to antibiotics. These lytic proteins could potentially replace or augment antibiotics in CDI treatment. We discuss candidate therapeutic lysins derived from phages/prophages of *C. difficile* and their potential as antimicrobials against CDI. Additionally, we review the antibacterial potential of some recently identified homologues of *C. difficile* endolysins. Finally, the challenges of endolysins are considered with respect to the development of novel lysin-based therapies.

## Introduction

*Clostridioides difficile* (formerly *Clostridium difficile*) is a Gram-positive, spore-forming obligate anaerobic bacillus, and the causative agent of healthcare-associated (HA) infectious diarrhea.^1^ In recent decades *C. difficile* associated infection has been associated with high morbidity and mortality, in particular in Europe, the USA, Canada, and Australia.^2^ Some reports recently also describe *C. difficile* infection (CDI) in Asia.^3,4^ There are nearly 462,100 CDI cases annually in the United States and at least 12,800 fatalities.^5,6^ In addition to loss of life, the treatment and management costs of CDI infection are significant, with an estimated annual cost of 800 USD million in the USA and €3,000 million in Europe.^7^ Although CDI cases in the United Kingdom decreased from 55,498 to 12,275 between 2007 and 2018,^8^ enormous effort has been put into CDI management strategies.^9^

CDI is mediated by up to three toxins; toxin A (enterotoxin), toxin B (cytotoxin) and the less common binary CDT toxin.^10^ These toxins damage the intestinal epithelium cell layer and activate the host inflammatory response that contributes to the disease pathology. The clinical symptoms range from mild to severe diarrhea and, to some instances, extend to potentially life-threatening conditions such as pseudomembranous colitis and toxic megacolon.^11^

The most common treatment strategy in CDI is to administer antibiotics such as metronidazole, vancomycin, and ﬁdaxomicin. However, recurrence of infection and treatment failure can occur.^12^ Recent reports suggest there is reduced susceptibility and resistance of *C. difﬁcile* toward these antibiotics.^13,14^ This has driven the exploration of alternative therapies to treat infections caused by this bacterium. Several treatment alternatives have gained traction and are at various stages of development; these include new antibiotics, probiotics, fecal microbiota transplantation (FMT), antimicrobial peptides, bacteriocins and phage therapy.^15–18^ The accessibility of phage genome databases and increased sequencing of phage have kindled interest in the application of phage encoded enzymes, especially endolysins, as alternative therapeutic agents.

Much has been written on the success of endolysins as targeted antimicrobials.^19–22^ A number of reviews have focused on the advantages, specificity and safety of endolysin therapy.^23–28^ In this work, we aim to review the existing literature on *C. difficile* endolysins and discuss their potency and their potential use as antimicrobials in the treatment of CDI.

## Antibiotic resistance in *Clostridioides difficile*

Generally, CDI is initiated following disruption of the normal intestinal microbiota by antibiotics that allow *C. difficile*, either native or acquired, to proliferate.^29^ The recommended antibiotics for primary and recurrent CDI are metronidazole, vancomycin and ﬁdaxomicin.^1,30^ Other antibiotics commonly used for bacterial infections such as cephalosporins, ampicillin, clindamycin, amoxicillin and ﬂuoroquinolones are also associated with a higher risk for CDI.^1,31^ The evolution of new ribotypes is often associated with acquisition of resistance as a result of inappropriate use of antibiotics.^14^ Many of the most common *C. difficile* epidemic ribotypes are associated with multidrug resistance.^14,31^ However, *C. difficile* is a spore-forming bacteria that can survive antimicrobial therapy and following germination relapse of CDI can routinely occur.

Several mechanisms of antimicrobial resistance have been identified in *C. difficile*. These include chromosomal resistance-associated genes, mobile genetic elements (MGEs), alterations in the antibiotic targets of antibiotics and/or in metabolic pathways, and biofilm formation. Examples of chromosomal resistance genes include those encoding β-lactamase-like proteins and penicillin-binding proteins (PBPs) that mediate resistance to the β-lactam antibiotics such as penicillin and cephalosporins.^14^ The *C. difficile* genome contains a wide range of mobile elements. MGE-like transposons facilitate the spread of antibiotic resistance genes by the process of conjugation, transduction, and/or transformation among *C. difficile* and/or between *C. difficile* and other bacterial species.^14^ Resistance to antibiotics of the macrolide-lincosamide-streptogramin B (MLSB) family, tetracycline and chloramphenicol in *C. difficile* is thought to be associated with different transposon families.^31^ Alterations in the antibiotic targets and/or in metabolic pathways is another important route of resistance development in *C. difficile*, and this mechanism mediates resistance to rifamycin, fluoroquinolones, metronidazole and vancomycin.^14,31^ Biofilms help pathogenic bacteria to survive unfavorable environmental stresses, including antibiotics.^32^ Biofilm formation is potentially involved in metronidazole and vancomycin resistance in *C. difficile*.^14,31^

## Bacteriophage endolysins

**Figure 1.**
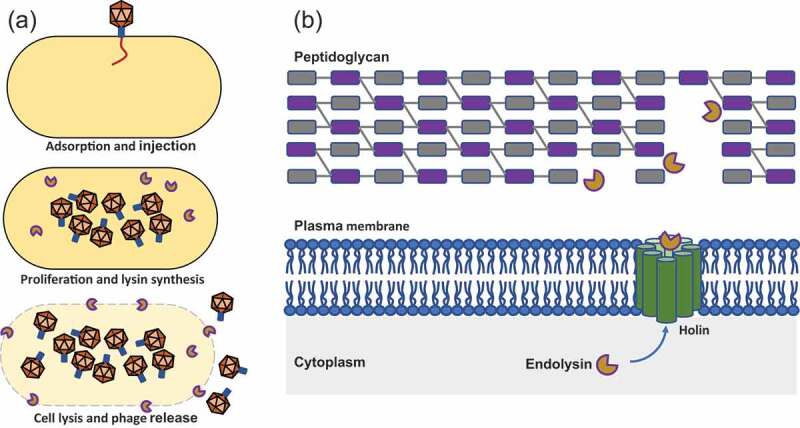
(a) Bacteriophage lytic cycle; (b) mode of action of endolysin against gram-positive cell walls of bacteria.

Bacteriophages or phages are viruses that infect and kill bacteria. Bacteriophages can adopt either of two life cycles, lytic and lysogenic. Both virulent and temperate phages may enter the lytic cycle, whereas only temperate phages utilize the lysogenic cycle.^33^ In most cases, the lytic cycle concludes with cell lysis that leads to cell death (Figure 1a). In single stranded DNA/RNA phages, the genome encodes a lysis effector which inhibits peptidoglycan (PG) biosynthesis from within the bacterium. On the other hand, double stranded DNA (dsDNA) phages utilize phage-encoded muralytic enzymes called endolysins (or lysins) that lead to cell envelope disruption at the final stage of phage reproduction. There are three different lysis mechanisms in dsDNA phages. The most studied and best understood mechanism is canonical lysis, where endolysins require the help of a second phage-encoded protein called a holin to act on the PG layer.^34^ Holin proteins accumulate and oligomerize in the cytoplasmic membrane (CM) in a time-controlled manner, and trigger depolarization and the formation of holes in the CM. This allows diffusion of endolysin to the membrane, facilitating the destruction of the PG layer (Figure 1b). The second mechanism requires a special class of holins, called pinholins, which form small, heptameric channels that help to depolarize the membrane. In the third mechanism, the lysis of the outer membrane of Gram-negative hosts is facilitated by spanin proteins.^35^ Spanins form a complex with outer membrane (OM) lipoprotein (o-spanin) and an integral cytoplasmic membrane protein (i-spanin) and disrupt the OM by enzymatic degradation, pore formation and inner membrane-outer membrane fusion.^36–38^

Endolysins are ‘enzybiotics’, a promising class of antibiotics derived from enzymes. Endolysins possess high speciﬁcity against the genus or species infected by the phage from which they were derived, which is believed to be one of their key advantages over classical wide-spectrum antibiotics. Thus, these lytic enzymes, specific for undesired pathogenic bacteria, rarely lyse non-target bacteria, including commensals of our microbiota or ‘good’ bacteria in foods (e.g. starter cultures) or those in the environment.^39,40^ The White house National Action Plan for Combating Antibiotic-resistant Bacteria has listed “phage-derived lysins to kill specific bacteria while preserving the microbiota” among the non-traditional therapeutics of note.^41^

It is possible to expand the lytic spectrum of an endolysin by the exchange or addition of certain domains beyond the native endolysin’s serovar, species, or even genus speciﬁcity.^42^ Endolysins can also act synergistically when used in combination with each other (i.e. two endolysins with different cleavage speciﬁcities) or with other antimicrobial agents.^20,43^ Other advantages of endolysins over traditional antibiotics are rapid host killing, low chance of resistance development, the potential to kill multi-drug-resistant bacteria, synergism with different antibacterial agents, and the ability to actively work in biofilms as well as on mucosal surfaces.^44–49^ Nowadays, the emergence of multidrug-resistant pathogens has revitalized the interest in alternative therapies. Due to the unique properties and advantages of endolysins over bacteriophages, endolysins are highly ranked alternatives in eradicating drug-resistant pathogens. Different properties such as specificity and host range,^39,50–52^ mode of action,^53–55^resistance development,^56,57^ stability,^58,59^ and pharmacokinetics^60,61^ between bacteriophage and endolysins are summarized in Table 1.

**Table 1.** Comparison of bacteriophages and endolysins as antimicrobials.

Selected properties	Bacteriophage	Endolysin
Specificity and host range	Generally specific to one bacterial species (or strains within a species). Limited impact on microbiota composition.	Narrow or broad depending on the chemical structure of the targeted macromolecule. Limited impact on microbiota composition.
Mode of action	Bacteriolytic activity depends on the titer, multiplicity of infection (MOI), burst size and propagation rate.	Bacteriolytic activity depends on concentration and minimum inhibitory concentration (MIC).
Resistance development	Resistance developed by mutation, receptor modification, passive adaptation, restriction-modification, CRISPR-Cas, pseudolysogeny.	Bacteria are less likely to develop resistance to endolysins.
Stability	Stability properties dependent on structural protein composition.	Endolysins have a short half-life, but effectively work in short duration due to the rapid mode-of-action.
Antibiofilm activity	Relatively effective with limited penetration capacity.	Effective against biofilms with higher penetration capacity.
Inflammatory response	Reticuloendothelial system (RES) clearance and immunogenic.	Immunogenic, induction of antibody production.
Pharmacokinetics	Not properly defined, self-replicating and can be cleared by immune system.	Evaluated in some endolysins; chemical structure affects penetration, plasma protein binding, and proteolysis degradation.
Combined therapy	Synergistic effect possible such as: phage cocktails, phage-protein and antibiotic–phage–protein combination.	Synergistic effect between two endolysins with different catalytic specificities or between an endolysin and an antibiotic.

## Basic structure and enzymatic activity of endolysins

Generally, endolysins have a conserved biological function directed at lysing infected bacterial cells. However, constant evolutionary pressure between bacteriophage and host bacteria has resulted in significant biochemical and structural variations among endolysins.^62^ Endolysins against Gram-positive and Gram-negative organisms are categorized differently due to the composition of the cell walls of their targets. Gram-positive endolysins are modular in structure with one or two N-terminal enzymatically active domains (EADs) and one or more C-terminal cell-wall binding domains (CBDs), these domains are usually connected by a short linker region.^62,63^ EADs contain the catalytic mechanism of endolysins, disrupting specific bonds within the bacterial peptidoglycan, whereas CBDs bind to constituents of cell walls to promote EAD localization to its target site and enhance catalytic efficiency. However, sometimes greater lytic activity has been observed in truncated endolysins containing only EADs.^20,64^ Gram-negative endolysins usually have single globular catalytic domain and lack a CBD.^65,66^ There have been some reports for Gram-negative endolysins that indicate a modular organization with a CBD at the N-terminus and EAD at the C-terminus which is inverse architecture typical to most Gram-positive endolysins.^67,68^

**Figure 2.**
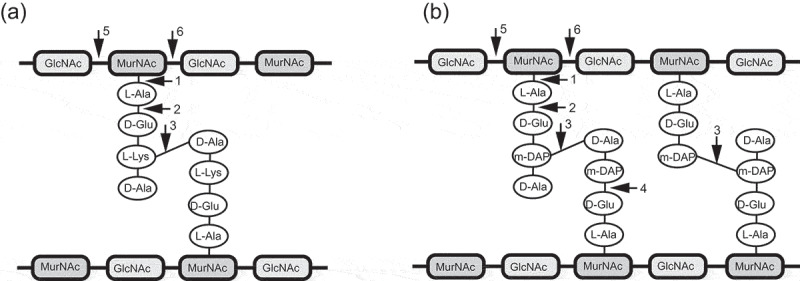
Schemetic presentation of bacterial peptidoglycan and generalized cut sites of peptidoglycan hydrolases. (a) A3α type peptidoglycan of *Staphylococcus aureus*; (b) A1γ type featuring peptidoglycan of *C. difficile*. The bonds potentially attacked by endolysins of different enzymatic specificities are indicated by numbers: 1) N-acetylmuramoyl-L-alanine amidases; 2) L-alanoyl-D-glutamate endopeptidases; 3) interpeptide bridge endopeptidases; 4) D-glutamyl-m-DAP endopeptidase; 5) N-acetyl-D-glucosaminidases; 6) N-acetyl-D-muramidases and lytic transglycosylases.

PG structure is highly conserved and consists of a polysaccharide of alternating N-acetylmuramic acid (MurNAc) and N-acetylglucosamine (GlcNAc) residues, linked by a β1-4 glycosidic bond (Figure 2). The D-lactoyl group of each MurNAc is linked with a short peptide stem, which is different between bacterial species.^69^ The tetrapeptide stem found in *C. difficile* is _L_-Ala-_D_-Glu-A2pm-_D_-Ala (A2pm: 2,6-diaminopimelic acid).^70^ Endolysins can recognize and digest a specific chemical bond within PG and are classified accordingly: (i) N-acetylmuramoyl-L-alanine amidases cleave the amide bond between N-acetylmuramoyl residues and L-alanine (the first amino acid of the peptide stem), (ii) L-alanoyl-D-glutamate endopeptidases target the bond between L-alanine and D-glutamate, (iii) interpeptide bridge endopeptidases digest the cross-link between peptide stems, (iv) D-glutamyl-m-DAP endopeptidase target bonds between D-glutamate and m-diaminopimelic acid, (v) N-acetyl-D-glucosaminidases hydrolyze the N-acetylglucosaminyl-β-1,4-N-acetylmuramine bond, (vi) N-acetyl-D-muramidases and lytic transglycosylases cleave the N-acetylmuramoyl−1,4-N-acetylglucosamine bond.

The chemotype of *C. difficile* PG is A1γ, where a *meso*-diaminopimelic acid (*meso*-A_2_pm) residue at position 3 of the peptide is directly cross-linked to a D-alanine at position 4 of the neighboring peptide. A similar structure is present in *Bacillus, Listeria* and some Gram-negative species.^40,70,71^ However, *C. difficile* also have a very high percentage of 3–3 peptide cross-links between two *meso*-diaminopimelic acid (*meso*-A_2_pm) residues^69^ (Figure 2b). Endolysin activity against these peptide cross-links has not yet been verified in *C. difficile*.

## *Clostridioides difficile* endolysins

**Figure 3.**
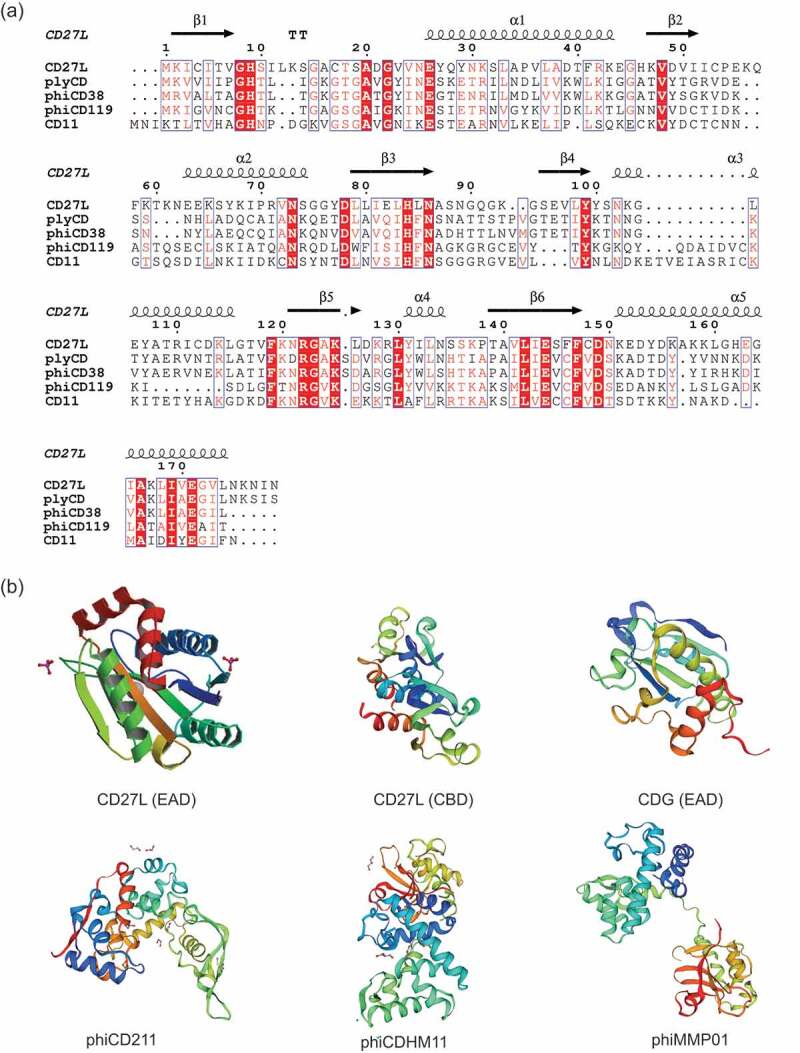
Sequence and structural analysis of *C. difficile* endolysins. (a) Sequence of the amidase containing catalytic domain of CD27L aligned to other amidase containing endolysins of *C. difficile*. Multiple-sequence alignment was performed by Clustal Omega and visualized by ESPript using CD27L endolysin structure (PDB code 3QAY) as the query. The position of amino acid residues based on CD27L amidase domain is shown. Secondary structure of CD27L_1–179_ is displayed, with arrows indicating beta strands and ribbons indicating alpha helices. Conserved residues can be visualized as white text on a red background, while amino acids with similar properties written in red text; (b) protein 3D structure of enzymatically active domains (EAD) and cell-wall binding domains (CBD). Homology modeling was performed by Swiss-model server (https://swissmodel.expasy.org/). The predicted model of CDG, phiCD211, phiCDHM11 and phiCDMMP01 were generated using protein data bank (PDB) templates 2L47, 5WQW, 4HPE and 4FDY, respectively.

The use of phage therapy in CDI is limited due to the temperate nature of *C. difficile* bacteriophages (reported to date). In most cases low titers of lysogenic phages have been recovered after induction with mitomycin C. These phages can easily reintegrate into the host genome following the removal of inducers.^72^ In the case of reported lytic phages, these may also have access the lysogenic life cycle.^73,74^ Efforts have focused on alternative options and the exploitation of *C. difficile* phage endolysins have sparked interest as therapeutic alternatives for CDI. Through published articles and online database searches, we identified sequences of putative endolysins from phage/prophage of *C. difficile*. The majority of these endolysins are amidases and hydrolases and are summarized in Table 2. The catalytic domains include amidase 3 domains, amidase 2 domains, glucosaminidases and NLPC_P60 domains. The sequence homology among the catalytic amidase domains was analyzed. A BLAST search for the sequence of the catalytic amidase domain of CD27L against the amidase containing endolysins obtained from *C. difficile* phage or prophage sequences was performed. After curation of the sequence cluster to remove duplicates, the sequences of five amidases (CD27L, phyCD, phiCD38, phiCD119 and CD11) were aligned and analyzed using ESPRIPT^90^ for sequence conservation. Although the sequence identity is low, several conserved residues were found. The conserved four amino acid residues (His 9, Glu 26, His 84, and Glu 144) are coordinating the zinc ion and responsible for catalytic activity (Figure 3a). The three-dimensional structures of major *C. difficile* endolysins (CD27L, CDG, phiCD211, phiCDHM11 and phiCDMMP01) have been predicted by homology modeling using SWISS MODEL, an online tool (Figure 3b). The potential templates for target endolysins were identified based on the sequence coverage and percentage of identity between the target and template sequence, except for CD27L whose EAD and CBD three-dimensional structure are already available (PDB code 3QAY and 4CU5). Due to the presence of different catalytic groups, the folding patterns are different in amidases, glucosaminidases and NLPC_P60 containing *C. difficile* endolysins.

**Table 2.** The list of endolysins of *C. difficile* phages/prophages.

Phages/Prophages	Putative endolysin (Name of ORF)	Domain and features	References
phiCD119	ORF35	MurNAc-LAA/Amidase_3	^75^
phiC2	phiC2p38 & phiC2p22	MurNAc-LAA/Amidase_3 & NLPC_P60/pfam00877*	^76,77^
phiCD27	gp34	MurNAc-LAA/Amidase_3	^78^
phiCD6356	phiCD6356_28	MurNAc-LAA/Amidase_3	^79^
phiCD38-2	gp23	MurNAc-LAA/Amidase_3	^80^
phiMMP02	gp34	MurNAc-LAA/Amidase_3	^72^
phiMMP04	gp26 & gp16	MurNAc-LAA/Amidase_3 & NLPC_P60/pfam00877*	^72^
phiCDMH1	gp34 & gp24	MurNAc-LAA/Amidase_3 & NLPC_P60/pfam00877*	^81^
phiCDHM13	gp25 & gp15	MurNAc-LAA/Amidase_3 & NLPC_P60/pfam00877*	^82^
phiCDHM14	gp25 & gp15	MurNAc-LAA/Amidase_3 & NLPC_P60/pfam00877*	^82^
phiCDHM19	gp40 & gp26	MurNAc-LAA/Amidase_3 & NLPC_P60/pfam00877*	^82^
phiCD211	PHICD211_20039 & PHICD211_20040	MurNAc-LAA/Amidase_3 & Glucosaminidase/pfam01832*	^83^
phiCDIF1296T	CDIF1296T_phi042 & CDIF1296T_phi043	MurNAc-LAA/Amidase_3 & Glucosaminidase/pfam01832*	^84^
phiCD24-1	PHICD2401_20030	MurNAc-LAA/Amidase_3	^83^
phiCD111	PHICD111_20024	MurNAc-LAA/Amidase_3	^83^
phiCD146	PHICD146_20023	MurNAc-LAA/Amidase_3	^83^
phiMMP01	PHIMMP01_20036 & PHIMMP01_20024	MurNAc-LAA/Amidase_3 & Glucosaminidase and NLPC_P60*	^72,83^
phiMMP03	PHIMMP03_20039 & PHIMMP03_20024	MurNAc-LAA/Amidase_3 & Glucosaminidase and NLPC_P60*	^72,83^
phiCD481-1	PHICD48101_20027	CwlA/Amidase_2	^85^
phiCD505	PHICD505_20034	MurNAc-LAA/Amidase_3	^85^
phiCD506	PHICD506_20027	MurNAc-LAA/Amidase_3	^85^
phiCDHM11	phiCDHM11_gp25 & phiCDHM11_gp15	MurNAc-LAA/Amidase_3 & NLPC_P60/pfam00877*	^86^
phiCDKM9	CDHM9_32	MurNAc-LAA/Amidase_3	^87^
phiCDKM15	CDKM15_37	MurNAc-LAA/Amidase_3	^87^
phiSemix9P1	Semix9P1_phi34	MurNAc-LAA/Amidase_3	^88^
*C. difﬁcile* 630	YP_001088405 & WP_009895119.1	MurNAc-LAA/Amidase_3	^20,89^
*C. difﬁcile* DA00211	EQH20562.1	CwlA/Amidase_2	^89^

*Present study.

### CD27L

The endolysin CD27L is derived from *C. difﬁcile* bacteriophage φCD27, a temperate bacteriophage belonging to the Myoviridae family with a genome length of 50,930 bp.^78^ This was the first *C. difficile* endolysin to be cloned and recombinantly expressed. The CD27L endolysin is 270 amino acids with an estimated molecular weight of ~30 kDa. CD27L is a modular endolysin that contains one EAD and one CBD. It has no transmembrane regions or signal peptide regions. The catalytic domain of CD27L belongs to the N-acetylmuramoyl-L-alanine amidase class (amidase 3). This lysin is active against a panel of 30 different *C. difficile* strains, including the hypervirulent ribotype 027, while a selection of the commensal bacteria of the GI tract are insensitive.^78^ Endolysin delivery to the gut environment is a challenge, and one approach is to use a genetically modiﬁed lactic acid bacterium. Crude protein extracts from *Lactococcus lactis* expressing CD27L have been shown to successfully lyse target cells to a similar degree to that observed for *E. coli* expressed lysin.^78^ It was found that CD27L lyses cells over a broad pH range, indicating it should remain active in the GI tract environment. Furthermore, the CD27L lysin catalytic domain, CD27L_1-179_, is more effective and exhibits a higher degree of specificity than the full-length endolysin.^64^ However, CD27L activity has not been tested an *in vivo* study.

### PlyCD

The lysin PlyCD is contained within a prophage in a multidrug resistance *C. difficile* strain, CD630.^20^ PlyCD is 262 amino acids with a molecular weight of ~28 kDa. The amino acid sequence identity between PlyCD and CD27L is 33%, with 34.6% identity between the catalytic domains. Both the full-length PlyCD and the recombinantly expressed truncated PlyCD_1-174_ are active against a number of *C. difficile* strains. Indeed, PlyCD_1-174_ has significantly greater lytic activity (>4-log kill) and a broader spectrum of activity while retaining high specificity toward *C. difficile* versus other commensal bacterial species. Additionally, a combination of PlyCD_1-174_ with a subinhibitory dose of vancomycin was significantly more successful *in vitro* against *C. difficile* compared to PlyCD_1-174_ or vancomycin alone.^20^ The functional capability of PlyCD_1-174_ was further confirmed by an *in vivo* mouse model study where the PlyCD treated mice showed increased survival and a delay in morbidity and mortality rate as compared to the buffer-treated control animals. Moreover, an *ex vivo* treatment model of mouse colon infection showed PlyCD_1–174_ functioned effectively in the presence of intestinal contents and significantly reducing colonizing *C. difficile* compared to buffer control.^20^

### CDG and CD11

The lysin proteins CDG and CD11 were identified in *C. difficile* DA00211 and *C. difficile* 630, respectively.^89^ Both lysins were expressed recombinantly and were found to be effective against *C. difficile* cells in a dose-dependent manner, reducing levels by up to four logs within five hours. These lytic proteins were active against clinical isolates of *C. difficile*, while no activity was observed against *Bacillus* or *Staphylococcal* species. The biocatalytic mechanism showed that these enzymes cleave bonds between N-acetylmuramoyl and L-alanine within the cell wall PG.^89^

### LHD

The modular structure of lysin proteins can facilitate the modification of bacteriolytic activity, speciﬁcity, solubility, and other physicochemical properties of these proteins in order to design novel antimicrobials. A lysin, LHD, was engineered to have the catalytic domain of a lysin protein from a *C. difficile* bacteriophage phiC2 fused with the functional domain of a human defensin protein HD_5_ by a 3-repeating unit linker; (GGGGS)_3_.^91^ The reason for choosing the catalytic domain of phiC2 is that phiC2 is present in the majority of human isolates of *C. diﬃcile*^92^ and it may have a wide spectrum of lytic activity. On the other hand, human defensin protein HD_5_ has been documented to inhibit hypervirulent *C. diﬃcile* strains.^93^ So, the hybrid would be more active than these individual antimicrobials. It was found that this lysin-human defensin fusion protein was active against several clinical *C. difficile* strains, including the epidemic 027, 078, 012, and 087 strains that are prevalent in many diﬀerent regions of world.^94^ The minimum inhibitory concentration (MIC) of LHD was lower than the MIC of the lysin protein LCD. The fusion protein was also active in a broader pH range (6.0, 7.0, and 8.0). In an *in vivo* mouse model, the LHD treated group had reduced symptoms mortality from CDI compared to the control buffer treated group. In addition, LHD significantly decreased the *C. difficile* spore count and toxin production in feces of the infected mice.^91^

### Cell wall hydrolases

Cell wall hydrolases (CWHs) are classified based on their origin as endolysins, exolysins and/or autolysins.^95,96^ Some of the *C. difficile* phage/prophages contain putative CWH sequences that may have potent lytic activity as endolysins (Table 2). The major catalytic domains present in *C. difficile* phage/prophage CWHs are NlpC/P60 and glucosaminidase. The NlpC/P60 domain is described as a superfamily and has a diverse range of catalytic activity including cleaving N-acetylmuramate-L-alanine linkages and the 4–3 linkage between D-Glu and *m*-DAP residues.^97–99^ The glucosaminadases hydrolyze the glycosidic bond of the sugar backbone. The putative N-acetylglucosaminidases (EC.3.2.1.96) that are present in *C. difficile* phages/prophages were also present in the prophage LambdaSa2 of *Streptococcus agalactiae* and exhibit β-D-N-acetylglucosaminidase activity.^100^

## Bacterial resistance to endolysins

Bacteriophage endolysins have a unique attribute in comparison to intact phages and antibiotics, in that resistance development is an extremely rare event. Generally, antibiotics work by inhibiting essential metabolic pathways of bacteria leading to cell death.^101^ However, bacteria have found ways to overcome this adverse situation by using alternative metabolic pathways. It is difficult for bacteria to find means of resistance to endolysin as they bind to and degrade highly conserved peptidoglycan targets within the cell wall.^102^ Any mutations leading to endolysin resistance would be damaging to the integrity of the cell and thus a very rare event.^103^ Although no attempts have been made to study lysin-resistance development in *C. difficile*, there are some studies using other bacterial strains that have investigated repeated lysin exposure and revealed resistance did not develop to either native or engineered phage lysins.^40,104-106^ Some CWHs of *C. difficile* have more than one catalytic domain and this theoretically lowers the chance of mutation in multiple target sites in bacteria. Similar observations have been made with *S. aureus* endolysins.^107^

## Safety and current trials

Bacteriophages are naturally an integral part of the human microbiota and thus the release of phage-derived lysins is unlikely to have a harmful effect on human health.^108^ The safety of phage lysins has been confirmed in a number of animal model systems.^20,47,109,110^ The impact of lytic proteins on inflammatory responses and/or their toxicity has been evaluated in animal models and it was observed that the administration of some lysin proteins, for example, Cpl-1 and MV-L, triggered an immune response which ultimately resulted in the production of antibodies against this protein.^111,112^ In another study, low levels of antibodies and/or cytokine production were observed in animals compared with untreated controls following endolysin treatment.^113,114^ Despite the number of animal trials published, only a few lysins have undergone human clinical trials. SAL200 is the first endolysin-based therapeutic formulation against MRSA. It is derived from *Staphylococcus* phage SAP-1 that infects *Staphylococci*, including MRSA and vancomycin-resistant *S. aureus* (VRSA) strains. Recently, protein SAL200 was evaluated in humans by intravenous infusion as part of a phase 1 clinical trial. Single ascending intravenous doses (0.1 mg/kg to 10 mg/kg) were applied to healthy male volunteers in order to assay pharmacokinetics, pharmacodynamics, and tolerance of SAL200.^61^ No serious adverse effects as well as recurrence of infection were observed in volunteers except more than three participants noticed mild and temporary effects like fatigue, headaches and myalgia. Another endolysin-based product called Staphefekt SA.100, developed by Dutch biotech company Micreos, has been available in Europe since 2017 for human use. Staphefekt SA.100 is an engineered chimeric endolysin for topical skin application that specifically binds to the cell wall of *S. aureus* and cleaves the cell membrane via endopeptidase and putative amidase activities.^115^ In a case study on three human subjects with chronic and recurrent *S. aureus*-related dermatoses showed that Staphefekt™ improved the clinical symptoms, but they rapidly recurred if the treatment was ceased. Potentially due to the recolonization of *S. aureus* from the nose and environment. It has also been shown that the long term daily use of Staphefekt did not result in generation of bacterial resistance during chronic and recurrent *S. aureus* treatment.^22^ A multi-center, placebo-controlled, double-blinded and randomized superiority trial study (ClinicalTrials.gov, NCT02840955) of Staphefekt showed application on the skin, targeting only *S. aureus* and leaving skin commensals unharmed, improves *S. aureus*-related skin infections, such as eczema, acne, and rosacea.^116^ Staphefekt is registered as a (class 1) medical device in Europe and available as an over-the-counter treatment in the form of a cream or gels. There are several other products at different stages of clinical trials, some with promising results that will pave the way for future endolysin-based therapies.^107,117^

## Challenges of endolysin therapy in CDI

There are several challenges facing the commercialization of endolysins, for instance, large-scale production and formulation, targeted delivery and regulatory framework amongst others. Until now, the use of endolysins as human therapeutics has not been approved in the United States or Europe, except for the endolysin-based product Staphefekt which is marketed in the EU as a ‘medical device’.^22^ Although the role of phage-based lysins is already established and examined in different animal infection models, the study of *C. difficile* endolysins is still limited. It may be due to some experimental limitations, particularly the development of targeted enteric delivery of such endolysins. To date, only two studies were performed where *C. difficile* lysins were examined in animal models for clinical development.^20,91^ In both studies, the targeted delivery of endolysins to the gut was by direct administration via oral gavage. Lysins are non-replicating protein molecules, with short *in-vivo* half-lives.^33,118^ The enteral delivery of phage lytic proteins faces the challenge of maintaining enzyme activity at low pH and in the presence of proteolytic enzymes of the stomach. To avoid such obstacles, the development of novel delivery strategies is important. Nanoparticle-based anticancer drug delivery to eukaryotic cells are becoming more popular as a treatment strategy.^119,120^ It also offers possibilities to effectively target bacterial cells.^121,122^ Endolysins could also be encapsulated in polymeric nanoparticles to give protection from the harsh gastric environment. Another proposed innovative approach is to deliver and preserve lysins in the gastrointestinal tract via engineered lactic acid bacteria that actively excrete the endolysin during gut transit.^123,124^ The *C. difficile* phage endolysin CD27L has been successfully expressed in *Lactococcus lactis* MG1363.^78^ Additionally, the probiotic strain *Lactobacillus johnsonii* FI9785 was precisely engineered to deliver and secrete an endolysin active against *C. perfringens*.^124^

## Conclusion

*Clostridioides difficile* is recognized as the leading cause of nosocomial and community-acquired diarrhea associated with exposure to antibiotics. Antibiotic resistance and a high rate of recurrence limit the usefulness of current antimicrobials used for primary *C. difficile* infection; alternative solutions are urgently required that work effectively while maintaining the gut microbiota. Phage lytic proteins show great potential in this regard as a replacement for, or as an additional therapy to, the use of traditional antibiotics in CDI. In fact, several endolysins active against *C. difficile* are currently being investigated in this regard. Major features, such as high catalytic activity, modular structure, and the possibility of engineering, support the development of these novel alternatives to conventional antibiotic therapy. The possibility of resistance development to phage lytic proteins remains a real possibility worthy of further investigation. To date, preclinical and clinical studies demonstrate that endolysins are safe and very effective antimicrobials.
